# Regenerative Therapy in Comprehensive Oral Rehabilitation for Pediatric Osteogenesis Imperfecta: A Case Report

**DOI:** 10.7759/cureus.76982

**Published:** 2025-01-05

**Authors:** Mridula Goswami, Farheen Sultan, Archana Singh

**Affiliations:** 1 Pediatric and Preventive Dentistry, Maulana Azad Institute of Dental Sciences, New Delhi, IND

**Keywords:** brittle bone disease, endovital therapy, full mouth rehabilitation, pediatric dentistry, regenerative endodontic therapy

## Abstract

Osteogenesis imperfecta (OI) is a rare genetic condition marked by brittle bones and various degrees of skeletal deformity. It is also referred to as brittle bone disease. There are four forms of OI, and its management relies on preventing fractures and increasing bone strength, which becomes more difficult when treating a juvenile patient in a dental setting. A six-year-old child with OI, type 1, with a history of recurrent multiple fractures, complained of pain in his mandibular teeth. The patient was receiving biannual bisphosphonate injections for OI. During the initial dental appointment, the patient was apprehensive and afraid, but cooperative behavior was achieved using non-pharmacological behavior management techniques. Full mouth rehabilitation was done while restoring the vitality of carious permanent teeth using regenerative endodontic therapy. The patient was then followed up on a regular basis to ensure progressive apical root closure. Regenerative endodontics uses the concept of tissue engineering to restore the root canals to a healthy state, allowing for the continued development of the root and surrounding tissue. This case report discusses the issues in managing a pediatric patient with OI as well as the benefits of regenerative endodontic treatment therapy in preserving tooth vitality.

## Introduction

Osteogenesis imperfecta (OI), commonly known as brittle bone disease, is a congenital condition marked by increased bone fragility and reduced bone mass. The majority of instances of OI are caused by mutations in the collagen 1 type 1 and collagen 2 type 2 genes, which encode the pro-alpha 1 and 2 polypeptide chains of type 1 collagen. According to the classification of Sillence et al., there are four forms of OI [1]. OI affects roughly one in every 10,000-20,000 persons worldwide. The prevalence rate of type 1 OI has been observed to range from 2.35 to 4.7 per 100,000 worldwide. The incidence of OI varies from approximately 1/15,000 to 1/20,000 in India [2]. The reported incidence of type 2 OI ranges from one in 40,000 to 1.4 in 100,000 live births. The exact occurrences of type 3 and 4 OI are unknown, but they are far less prevalent than type 1. The most affected tissues are ligaments, sclera, bone, and dentin [3]. Patients with OI frequently exhibit aberrant bone development, growth deficiency, bone fragility, blue sclerae, hearing loss, skin thinness, joint laxity, hypermobility, and dentinogenesis imperfecta [4]. Pediatric patients present with severe dental symptoms such as taurodontism (6-11%) [5], pulp obliteration (46.4%), and agenesis (23.9%) [6] that necessitate the immediate and preventive care of a pediatric dentist to understand and manage the issue. The vitality of the dentin-pulp complex is fundamental to the life of the tooth and is a priority for targeting clinical management strategies. One of the approaches to restoring tooth structure is based on biology: regenerative endodontic procedure. The basic rationale behind this approach is that patient-specific tissue-derived cell populations can be used to functionally replace integral tooth tissues. This case report emphasizes the need and complexities of dental management of such a situation in a pediatric OI case while being minimally invasive using the regenerative endodontic procedure.

## Case presentation

**Figure 1:**
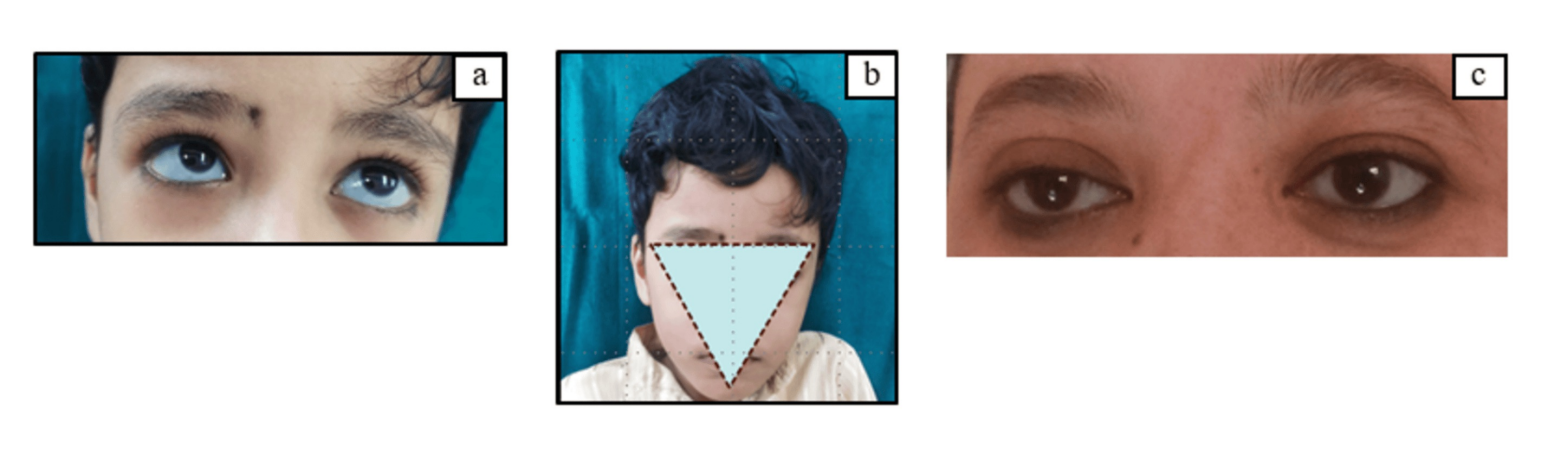
(a) Evident blue sclera. (b) Triangular shape of the face. (c) Blue sclera of the mother

A six-year-old male child along with his parent presented to the OPD of the Department of Pediatric and Preventive Dentistry with a chief complaint of pain in the lower left back tooth region for the past four days. Afterward, he started experiencing pain in the lower left back teeth region. The pain was continuous, dull aching, nocturnal, and non-radiating in nature which was not relieved by medications. The patient has a diagnosed history of OI (type 1) and receives an infusion of biannual calcium, vitamin D, and bisphosphate (pamidronate). Additionally, he had a history of three episodes of bone fractures (twice in the humerus and once in the clavicle) at different time periods within a span of three years. Family history revealed that the patient's mother also had OI having blue sclera and triangular face shape with bone fragility (Figure 1).

**Figure 2:**
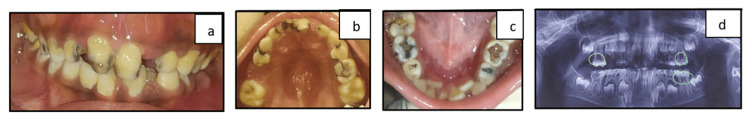
Preoperative images (a) Frontal view showing carious and retained 52, 61, and 62 (b) Maxillary occlusal view showing caries wrt 51, 52, 54, 61, 62, 64, 65, 16, and clinically missing 55 (c) Mandibular occlusal view showing caries wrt 74, 84, 75, 85, and 46 (d) Orthopentogram revealing the preoperative image of 46 and hypotaurodontism wrt 16, 26, and 36 wrt: with respect to

During the intraoral examination, tooth 46 was grossly carious, tender on percussion, and non-responsive to cold test with periapical radiolucency along with an open apex present as seen in the orthopentogram (Figure 2). Keeping the patient's medical condition in mind, we planned for a minimally invasive treatment in order to restore the vitality of the tooth; thus, the regenerative endodontic treatment was done in the present case.

During the first appointment, rubber dam isolation followed by access opening after the administration of 2% lignocaine was done in 46. Working length determination was performed, followed by the biomechanical preparation of the mesiobuccal and mesiolingual canals using the hand ProTaper system up to size #F3. 1% hypochlorite was used to irrigate the canal, calcium hydroxide dressing was placed after drying the canal using paper points, and temporary restorative material was placed. The patient was then again recalled after three weeks.

**Figure 3:**
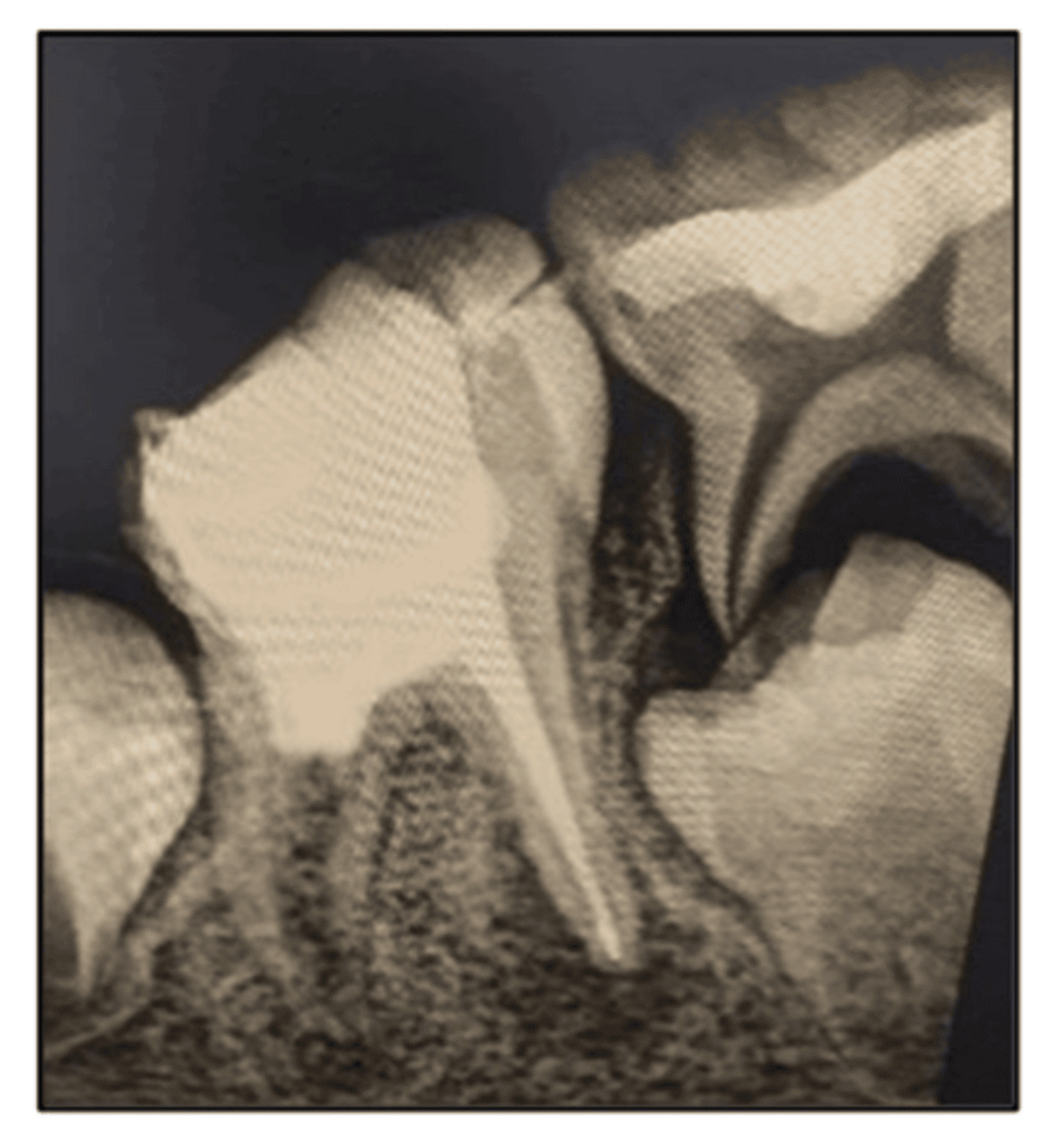
RVG wrt 46 showing the mesial canals obturated using gutta-percha and revascularization using MTA done in the distal canal RVG: radiovisiography; wrt: with respect to; MTA: mineral trioxide aggregate

During the second appointment, the patient was asymptomatic. After the administration of local anesthesia without vasoconstrictor and rubber dam isolation, the distal canal of tooth 46 was irrigated using ethylenediaminetetraacetic acid. Bleeding was induced using a pre-curved K-file rotating in a circular motion 2 mm beyond the apex, and it was irrigated with normal saline. Bleeding was induced again and was stopped, followed by the placement of 3-4 mm of mineral trioxide aggregate (MTA) (Angelus Dental Solutions, Londrina, Paraná, Brazil) in the canal above the formed clot. A thin layer of glass ionomer cement was placed over the MTA. The mesial canals were obturated using gutta-percha. The tooth was restored with composite restoration (Tetric N-Ceram, Ivoclar, Schaan, Liechtenstein) (Figure 3). 

**Figure 4:**
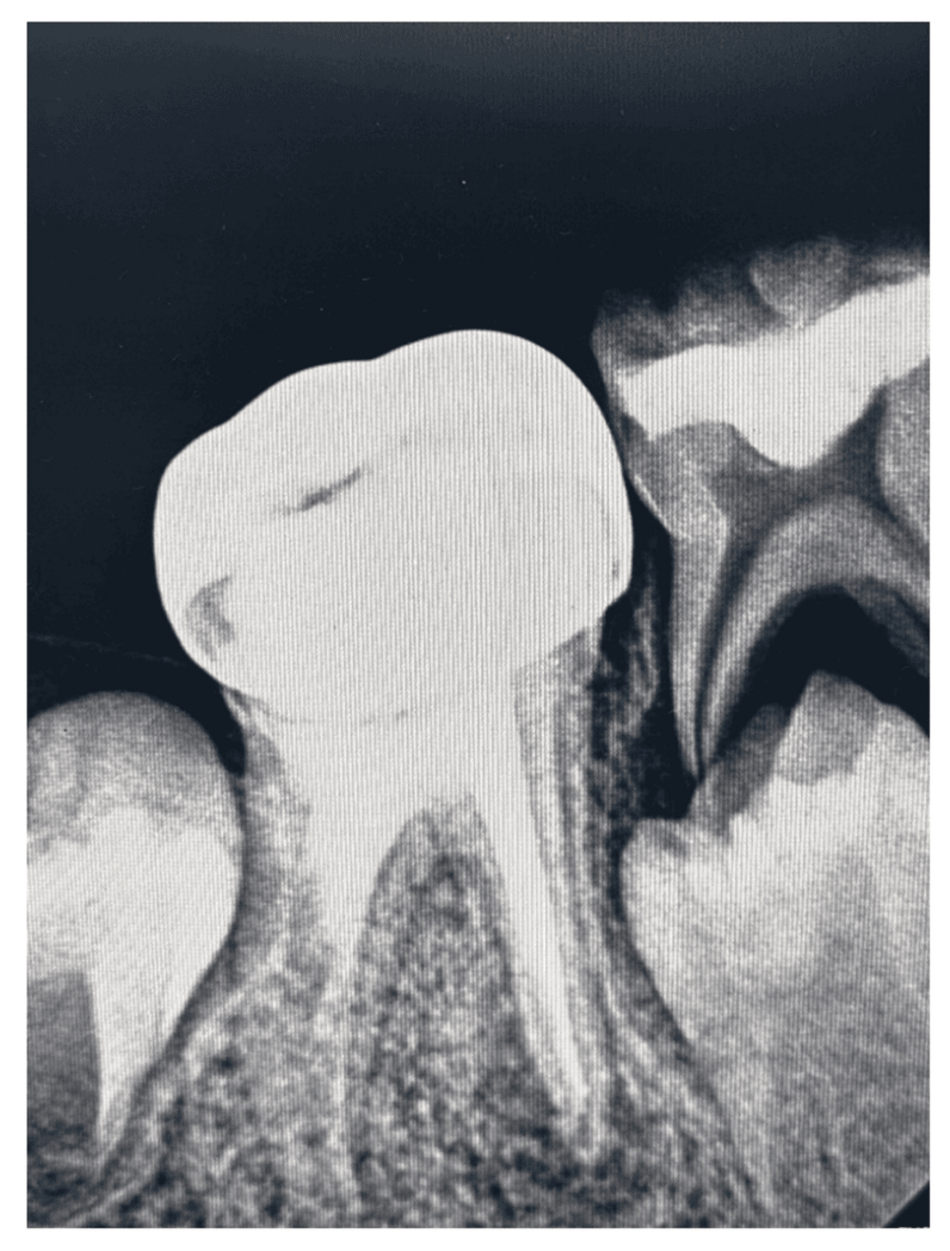
RVG wrt 46 showing the mesial canals obturated using gutta-percha and revascularization using MTA done in the distal canal followed by stainless steel crown RVG: radiovisiography; wrt: with respect to; MTA: mineral trioxide aggregate

During the third appointment, the patient was recalled after two weeks and was asymptomatic. A stainless steel crown was placed after tooth preparation in order to provide full coverage protection (Figure 4).

**Figure 5:**
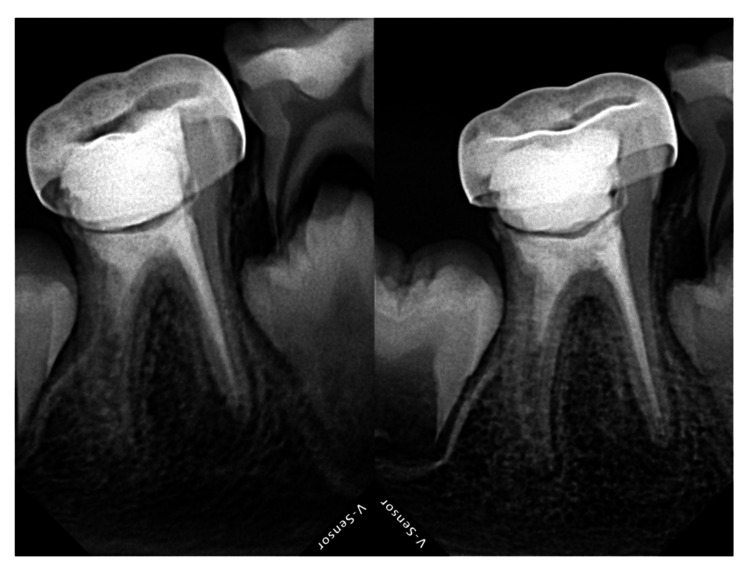
(a) RVG wrt 46 showing three months follow-up of tooth with stainless steel crown showing evidence of apical root closure in the distal canal and healed periapical lesion. (b) RVG wrt 46 showing eight months follow-up of tooth with stainless steel crown showing evidence of apical root closure in the distal canal and healed periapical lesion RVG: radiovisiography; wrt: with respect to

The patient has been scheduled for regular follow-ups and is currently asymptomatic after eight months with satisfactory healing and root formation observed in 46 (distal canal) (Figure 5a, 5b).

## Discussion

OI is a genetic disorder primarily characterized by fragile bones, often resulting in frequent fractures and skeletal deformities. It can affect dental development, leading to dentinogenesis imperfecta, where teeth are misshapen and exhibit discoloration and increased susceptibility to wear and breakage. Sillence et al. [1] classified it under four forms (Table 1).

**Table 1:** Clinical types of osteogenesis imperfecta Reference: [1]

Clinical types of osteogenesis imperfecta	
Osteogenesis imperfecta: type 1	Osteogenesis imperfecta tarda
	Osteogenesis imperfecta with blue sclerae
	Gene map locus 17q21.31-422, 7q22.1
Osteogenesis imperfecta: type 2	Osteogenesis imperfecta congenita (neonatal, lethal)
	Vrolik type of osteogenesis imperfecta
	Gene map locus 17q21.31-q22, 7q22.1
Osteogenesis imperfecta: type 3	Progressively deforming, normal sclerae
	Gene map locus 17q21.31-q22, 7q22.1
Osteogenesis imperfecta: type 4	Osteogenesis imperfecta with normal sclerae
	Gene map locus 17q21.31-022

Addressing these dental challenges necessitates comprehensive dental rehabilitation to enhance both function and aesthetics while considering the unique complexities associated with the condition [7]. Full mouth rehabilitation in patients with OI presents distinct challenges due to the systemic nature of the condition, characterized by brittle bones and dental anomalies. It affects the structural integrity of bones, including those in the jaw, and can manifest as dental problems such as tooth wear, malocclusion, and dental fragility [8]. In this present case report, meticulous patient evaluation, including medical history and assessment of bone fragility, was imperative to minimize the risk of fractures during dental intervention. Effective behavior management was the crucial part, especially considering the child's fearfulness stemming from his prior experience with multiple hospital visits. 

Given the patient's high caries index as per the Decayed, Missing, and Filled Surfaces (DMFS) score, restoration of carious teeth was done using giomer. Clinical studies suggested that giomer, being a recent advancement in fluoride-releasing, exhibits comparable morphology, marginal adaptation, and postoperative sensitivity similar to resin composites [9].

Regenerative endodontics can be viewed as an extension of the principles of minimally invasive dentistry (MID). The four pillars at molecular levels are bioactive materials, stem cells, growth factors, and their synergistic effects which align with the MID principles by aiming to preserve the natural tooth structure. Stem cells from the dental pulp, periodontal ligament, or surrounding tissues are recruited and stimulate odontoblast differentiation, and growth factors bind with their receptors for intercellular pathway signaling to promote regeneration. The extracellular matrix helps guide cell behavior and tissue development, and the bioactive materials provide calcium hydroxide and tricalcium phosphate to enhance mineralization and support dentin formation.

Utilizing techniques like regenerative endodontics with MTA not only promotes healing and continued root development but also helps in conserving the weakened tooth structure in such patients. Other alternative materials are being used as well such as Biodentine (Septodont, Saint-Maur-des-Fossés, France) and EndoSequence® (Brasseler, Georgetown, Georgia, United States). A prospective study done by Koli et al. [10] concluded that a combination of non-surgical endodontic treatment and vital pulp therapy is a viable biologically based minimally invasive treatment option for multirooted mandibular teeth.

In this case, we prepared an advanced form of treatment based on the patient's health state. With regenerative therapy in the distal canal and gutta-percha obturation in the mesial canals of tooth 46, we aimed to preserve the strength and restore the vitality of tooth structure which was the need of the time considering the patient's age and medical conditions. The mesial canals exhibited clinical tug-back, which was also checked by radiographic evaluation. This confirmed adequate apical fit, leading to gutta-percha obturation of the mesial canals to achieve a proper seal. This approach not only reduces the risk of postoperative complications but also preserves the natural dentition, which is crucial for proper masticatory function and aesthetics.

The American Association of Endodontists (AAE) clinical considerations for regenerative endodontic procedures define success by three measures [11]: The primary goal (essential) is the elimination of symptoms and the evidence of bony healing. The secondary goal (desirable) is increased root wall thickness and/or increased root length. The tertiary goal is a positive response to vitality testing. In the present case report, the primary goal was achieved successfully immediately after regenerative endodontic treatment. After eight months of follow-up, the patient showed successful periapical healing with no clinical signs and symptoms. The radiograph showed evidence of apical root formation in the distal root. The patient is under regular follow-up for the secondary goal (apical root formation) and tertiary goal (positive vitality testing). Long-term management of OI patients undergoing full mouth rehabilitation involves regular follow-up in addition to preventive measures to ensure optimal outcomes, maintain oral health, and prevent complications.

Clinical significance

Children with OI should be evaluated by a pediatric dentist as soon as the teeth begin to appear. Pediatric dentistry has seen remarkable strides with the advent of minimally invasive techniques and advanced materials such as MTA. Regenerative endodontics aims to restore vitality by utilizing stem cells, growth factors, and bioactive materials, maintaining tooth structure, relieving pain, and promoting healing through its conservative approach. This technique is employed in the present OI case where a more conservative treatment modality based on the concepts of MID coupled with behavior management is needed. Counseling of the child and parent helps in addressing emotional, behavioral, and social issues, thus enhancing the quality of life.

## Conclusions

Full mouth rehabilitation of patients with OI requires a comprehensive and multidisciplinary approach including the pediatric dentist, physician, and medical practitioner. Regenerative endodontics, with its minimally invasive techniques and focus on preserving tooth structure and pulp vitality, plays a crucial role in managing dental issues in these patients effectively. There are a few limitations such as technique sensitivity, longer appointments, and long-term follow-up in order to trace the prognosis of treatment.
